# Correlative studies on vitamin D and total, free bioavailable testosterone levels in young, healthy men

**DOI:** 10.1038/s41598-021-99571-8

**Published:** 2021-10-12

**Authors:** Anna Książek, Marek Mędraś, Aleksandra Zagrodna, Małgorzata Słowińska-Lisowska, Felicja Lwow

**Affiliations:** 1 Department of Biological and Medical Basis of Sport, University School of Physical Education, Wroclaw, Poland; 2 Department of Massage and Physical Therapy, University School of Physical Education in Wrocław, Wroclaw, Poland

**Keywords:** Physiology, Endocrinology

## Abstract

The relationship between vitamin D levels and testicular hormonal function in men has not been clearly established. Therefore, we aimed to investigate the relationship between deficiency/insufficiency levels of 25(OH)D and luteinizing hormone (LH), follicle-stimulating hormone (FSH), total (TT), free (FT), and bioavailable testosterone (BT), and sex hormone binding globulin (SHBG) in young, healthy men. We enrolled 176 healthy, active young men aged 18–35 years from a genetically homogenous population of Lower Silesia, Poland. Serum levels of 25(OH)D, LH, FSH, and TT were measured by electrochemiluminescence (ECLIA). FT levels were measured by enzyme-linked immunosorbent assay (ELISA). BT levels were calculated from TT, SHBG, and albumin. SHBG was measured by chemiluminescent immunoassay CLIA. We did not find any significant differences between the mean hormonal values (LH, FSH, TT, FT, BT, and SHBG) and the status of 25(OH)D level (deficient and insufficient). Based on our results, we concluded that there is no relationship between deficient and insufficient 25(OH)D concentration and androgen levels in young, healthy men.

## Introduction

Vitamin D deficiency is becoming a public health problem in all age groups in developing and developed countries^1^. From an evolutionary point of view, vitamin D is one of the oldest biologically active compounds. It has had various metabolic functions throughout the history of the species’ development, in different cells. This is echoed by the presence of its receptors in many tissues, organs, and systems, such as the skeletal muscles^2^, the thyroid gland^3^, the cardiovascular system^4^, the immune system^5^, and the reproductive system^6^.

Vitamin D is not actually an amine (as the name might imply), and its molecule is cyclopentanoperhydrophenanthrene, with a broken B ring. The biological action of vitamin D takes place in cells via specific receptors and genomic and extragenic mechanisms^7^. Vitamin D is assumed to affect the function of approximately 3% of the human genome by modulating tissue metabolism^8^. Vitamin D has been suggested to play a role in sex steroid production, based on the evidence of the expression of vitamin D receptors (VDR) and vitamin D metabolizing enzymes, which are simultaneously expressed in Leydig cells and also in Sertoli cells, germ cells, spermatozoa and in the epithelial cells lining the male reproductive tract^6,9^. Testosterone production, by the Leydig cells that reside in the interstitial compartment, is under strict control of LH, which induces steroidogenesis by increasing cyclic AMP production and the intracellular concentration of calcium ions (Ca^2+^) in Leydig cells and 1α,25-dihydroxyvitamin D might exert an influence by modulating this calcium-dependent LH response^10^. A possible mechanism for the effect of vitamin D on testosterone production might be indirectly hypothesized from the results of an in vivo study in vitamin D-depleted and vitamin D-repleted chickens, investigating on the testis expression of calbindin-D28K, a cytosolic calcium-binding protein involved in the regulation of intracellular calcium homeostasis, and proposed to be involved in testis hormone production in rats^6^.

The active biological form of vitamin D (known as calcitriol) is synthesized starting in the skin, with the initial synthesis of pre-vitamin D_3_ through a nonenzymatic reaction initialized by UV light, using 7-dehydrocholesterol^7^. As a part of vitamin D synthesis, human skin also expresses genes which encode enzymes participating in sequential cholesterol metabolism leading to pregnenolone and to corticosteroids. Androgen production, activation and metabolism also occurs in the skin, acting as an important site for the entire process. These molecules are involved in intra-, auto- or paracrine fashion to adjust local homeostasis. The skin can produce signals to induce rapid (neural) or slow (humoral or immune) effects at the local and systemic levels. These local and systemic responses are coordinated as mediated by the skin neuroendocrine system, employing local equivalents of the hypothalamo-pituitary-adrenal axis (HPA)^11,12^. It has been proved that, in response to a variety of stressors, skin can produce numerous hormonal elements which are expressed in systemic responses to stressors in the environment. Furthermore, it has been shown that the key corticosteroid synthesis enzymes resulting in dermal corticosterone and cortisol production are expressed in the skin^13^. Human exposure to sunlight led to increased vitamin D serum levels, UVR-mediated central HPA axis activation may be used as a rational basis for phototherapy in systemic autoimmune disorders, as well as different pathologies^12–14^.

Vitamin D exhibits features of endocrine action in the regulation of phosphate and calcium and parathormone metabolism, whereas it shows signs of an auto-, para- or juxtacrine signalling molecule in the remainder of its biological effects. With these mechanisms, it can affect the function of many cells, tissues, and systems, including Leydig cells^15^, which are the main source of testosterone in the male body.

It should be emphasised that the influence of vitamin D (mainly auto- and paracrine) is usually local and that studying them requires tissue analysis. The local effects of this type are generally not discernible at the blood serum level (they are not endocrine in the classic sense, and therefore do not affect the entire organism). Exceptions may include situations where there is a high level in a specific substrate or a specific tissue, such as adipose tissue.

Testosterone is the primary androgenic–anabolic hormone in the male body, the role of which is well known and unquestioned. Vitamin D, on the other hand, is receiving increased interest and is the subject of numerous studies with inconclusive results. This raises the question of whether vitamin D levels can modulate testosterone levels in men, and thus the functioning of the hypothalamic–pituitary–gonadal axis (HPA). The answer is currently unknown.

The aim of our study was to evaluate the effects of different 25(OH)D levels (deficient and insufficient) on the functioning of the HPA, i.e. on the levels of luteinizing hormone (LH), follicle-stimulating hormone (FSH), total testosterone (TT), free testosterone (FT), bioavailable testosterone (BT), and sex hormone binding globulin (SHBG) levels in young, healthy men.

## Methods

### Study participants

The present study is a part of a project entitled Andrological Status of Young Men in Lower Silesia (AndroLS). We conducted the study among 176 healthy young men aged 18–35 years from a genetically homogenous population of Lower Silesia, Poland. All of the enrolled men had completed secondary school and were either continuing their education or had graduated from universities. The demographic characteristics, physical activity, smoking status, and alcohol consumption were collected through questionnaires. The baseline characteristics of the study groups are presented in Table 1. The study was conducted in the winter season (December–March) in Lower Silesia, which is situated at the latitude of 51° 10′ N. None of the subjects used any dietary supplements containing vitamin D and/or calcium. Participants who used sunbed were excluded from the study.

**Table 1:** Baseline characteristic of the study groups.

	25(OH)D ng/ml (n = 176)		
	< 10 n = 70	10–20 n = 77	> 20 n = 29
Age (years)	25.29 ± 3.80	24.55 ± 3.78	24.13 ± 3.64
Body mass (kg)	80.8 ± 11.12	78.5 ± 11.41	80.0 ± 10.21
Height (cm)	182.0 ± 6.01	182.1 ± 6.09	181.6 ± 8.4
BMI (kg/m^2^)	24.4 ± 3.02	23.6 ± 2.88	24.2 ± 2.4
WHR	0.91 ± 0.13	0.89 ± 0.12	0.94 ± 0.08
Current smoker, n (%)	14 (20.0)	9 (11.7)	2 (6.9)
Alcohol drinking^a^, n (%)	62 (88.6)	72 (93.5)	24 (82.8)
MET-total [MET-min/week]	4800 ± 3864	5384 ± 5171	6932 ± 4195

Values are expressed as frequency (%) or mean ± SD.

*BMI* body mass index, *WHR* waist–hip ratio, *MET-total* total energy expenditure.

^a^Beer, wine, or spirits in the amount equivalent ≥ 50 g ethanol per week.

### Recruitment procedure

The presented study is a part of project entitled ‘‘Andrologic Status of Young Men in Lower Silesia’’ (AndroLS), whose purpose was to evaluate the associations between a range of lifestyle factors (physical activity, diet, addictions) and the seminological/hormonal profiles of young men with unknown fecundity. Recruitment procedure has been described as in the earlier manuscripts^16–18^.

We announced the study through the following media: fliers; notices; messages via Facebook, Twitter, and Instagram; and personal communication to university students, societies, and clubs in the region. We directly contacted more than 5000 men^16–18^.

Those who responded to the invitations (n = 500) were asked to fill out the questionnaires covering medical history, nutritional habits (recall diary of the last 7 days), and physical activity (IPAQ, last-7-day recall)^19^. We did not enroll subjects who were being diagnosed or treated because of andrological pathology, who had undergone urogenital surgery, who had known or suspected fertility issues, who had chronic diseases, or who received medications that could interfere with hormonal evaluation. Although we did not offer any financial incentive to the volunteers, the benefit for those who decided to participate in the study was the knowledge of their andrological and nutritional status^16–18^.

From among 300 eligible men, we managed to gather blood samples and completed questionnaires. Eventually, all data was available for 176 subjects.

### Assessment of physical activity

The level of physical activity was evaluated using the International Physical Activity Questionnaire (IPAQ) Short Form^19^. The intensity of activity was defined as energy expenditure and was expressed in metabolic equivalents (METs): 1 MET is equivalent to the consumption of 3.5 ml of oxygen per minute per kg of body weight^20^. Physical activity estimated with the IPAQ was finally expressed in MET-minutes/week, being the sum of the individual energy expenditures during high-, intermediate-, and low-intensity activities^21^. The subjects were assigned to one of three levels of physical activity based on the IPAQ protocol. According to the IPAQ criteria, the physical activity of a person who achieves ≥ 600 or ≥ 3000 MET-min/week is classified as moderate or high, respectively. Those who do not meet the above-mentioned criteria were classified as physically inactive^19^.

### Blood testing

Blood sampling was carried out at 8.00 a.m. after 12 h of fasting and 24 h without physical activity. The serum was separated and stored at − 70 °C. The laboratory measurements of 25(OH)D_2_ and 25(OH)D_3_ (25(OH)D) were obtained by electrochemiluminescence (ECLIA) using an Elecsys system (Roche, Switzerland). For 25(OH)D, the intra- and inter-assay coefficients of variation (CVs) were 5.6% and 8.0%, respectively, and the limit of detection was 4 ng/ml (10 nmol/l).

The serum LH, FSH, and TT levels were measured by ECLIA (Cobas B 411 analyzer, Roche, Germany). Intra-assay and inter-assay CVs for LH, FSH, and TT were 2.0% and 5.3%, 1.8% and 5.2%, and 4.7% and 8.4%, respectively.

The serum FT level was determined by a commercial ELISA kit (NovaTec Immundiagnostica GmbH, Dietzenbach, Germany). Intra-assay and inter-assay CV for FT were 8.9 and 12.4%, respectively. The lowest detectable concentration of FT is 0.04 pg/ml.

The concentrations of BT was assessed with a calculator developed at the Hormonology Department, University Hospital of Ghent, Belgium (details on the calculation are available on the website http://www.issam.ch/freeresto.htm).

SHBG was measured by CLIA (Immulite 1000, Siemens, Tarrytown, NY, USA) with measurement range of 17.3–65.8 nmol/l and intra-assay and inter-assay CVs of 4.1% and 7.5% and 6.8% and 13.0%, respectively.

### Ethics statement

The study was approved by the Bioethics Committee of the University School of Physical Education, Wrocław, Poland (resolution number 36/2013) and all related procedures were conducted in compliance with the tenets of the Declaration of Helsinki regarding human subjects and the European Communities Council Directive of 24 November 1986 (86/609/EEC). Informed consent was obtained from all participants included in the study.

### Statistical analysis

The data were analysed with the statistics package of Sigmaplot version 13 (Systat Software) and the R environment (www.r-project.org). Descriptive statistics were used to summarise the baseline characteristic of the participants. Descriptive statistics are presented as mean ± SD in three strata, defined by the level of 25(OH)D: < 10 ng/ml, 10–20 ng/ml, and > 20 ng/ml (Table 1).

One-way ANOVA was used to compare unadjusted means of hormone levels (LH, FSH, TT, FT, BT, and SHBG) between the three categories. Vitamin D and hormone levels were assessed using analysis of covariance, after adjusting for age, BMI (body mass index), WHR (waist–hip ratio), alcohol intake, and cigarette smoking. The data are presented as mean ± standard deviation.

Pearson's correlation coefficient and partial correlation were used to evaluate the associations between 25(OH)D and hormonal parameters. Theory and an evaluation of the correlations between variables were used to identify potential confounding variables: age, BMI, WHR, alcohol intake and cigarette smoking.

## Results

This work is derived from a cross-sectional study of an initial population of 5000 young, healthy men from Lower Silesia (Poland). In our previous studies, we investigated in detail the overall quality of semen^16^ and its associations with physical activity^17^ and vitamin D concentration^18^.

The values of the mean total energy expenditure for the individual 25(OH)D concentrations were as follows: 4800 ± 3864 MET-min/week for the < 10 ng/ml group, 5384 ± 5171 MET-min/week for the 10–20 ng/ml group, and 6932 ± 4195 MET-min/week for the > 20 ng/ml group. It was found that according to the IPAQ criteria 6.3% of the participants had a total energy expenditure of < 600 MET-min/week, 35.5% were in the range of > 600 to < 3000 MET-min/week, and 60.2% had a total energy expenditure of > 3000 MET-min/week^19^.

Assuming serum 25(OH)D levels in the range of 30–50 ng/ml to be the physiological norm^22,23^, only 4% of the men in the study (n = 7) achieved this level. We found that 44% of the participants (n = 77) had a 25(OH)D concentration between 10 to 20 ng/ml, and 40% of them (n = 70) below 10 ng/ml, which is defined as vitamin D deficiency^23^ (Table 2).

**Table 2:** Adjusted mean (s.e.) of hormonal parameters per status of serum vitamin D level.

	25(OH)D ng/ml (n = 176)			p
	< 10 n = 70	10–20 n = 77	> 20 n = 29	
LH (mIU/ml)	5.09 ± 2.26	4.84 ± 2.29	5.28 ± 2.83	0.656
FSH (mIU/ml)	4.55 ± 3.59	4.16 ± 3.8	4.62 ± 6.21	0.807
TT (ng/ml)	5.99 ± 2.09	6.02 ± 1.91	6.03 ± 2.46	0.994
FT (pg/ml)	14.77 ± 15.11	15.11 ± 5.03	15.78 ± 8.57	0.771
BT (ng/ml)	2.51 ± 1.16	2.60 ± 0.91	2.50 ± 1.2	0.842
SHBG (nmol/l)	41.08 ± 19.21	37.73 ± 13.71	43.02 ± 17.85	0.266

Adjusted for age, BMI, WHR, alcohol intake and cigarettes smoking.

Table 2 presents the differences between the three study groups (categorised according to 25(OH)D levels) in terms of hormonal parameters. There was no statistical difference between the mean hormonal values (LH, FSH, TT, FT and BT, or SHBG) in the different categories of 25(OH)D.

The values of the Pearson coefficient and partial correlation after adjusting for age, BMI, smoking and alcohol intake are given in Table 3. According to our results, there was no statistically significant correlation between 25(OH)D and LH, FSH, TT, FT, BT and SHBG in studied group.

**Table 3:** Pearson’s and partial correlations between 25(OH)D levels and hormonal parameters.

	25(OH)D (ng/ml) (n = 176)			
	Pearson’s r	p	Partial correlation^a^ r	p
LH (mIU/ml)	0.065	0.393	0.078	0.308
FSH (mIU/ml)	− 0.037	0.624	0.006	0.941
TT (ng/ml)	− 0.005	0.6952	− 0.032	0.647
FT (pg/ml)	0.055	0.472	0.014	0.860
BT (ng/ml)	− 0.050	0.508	− 0.113	0.140
SHBG (nmol/l)	0.093	0.221	0.114	0.139

^a^Adjusted for age, BMI, WHR, alcohol intake and cigarettes smoking.

## Discussion

The primary source of testosterone in men is steroidogenesis in the Leydig cells, regulated by LH secretion in a negative feedback mechanism. Peripheral testosterone synthesis in men (as opposed to women) has little systemic significance, although it may exert local effects on metabolic processes related to, for example, the general anabolic condition of the body.

In a healthy man, increases in blood testosterone levels can occur through two primary physiological mechanisms:

1. under the effect of increased LH concentration (hypothalamic–pituitary influence)—then the serum concentration of LH and testosterone increase
2. as a direct result of factors that stimulate the steroidogenesis process in the gonads—then there is an increase in serum testosterone levels and a reduction in LH levels.

Of course, there may be an increase in serum testosterone as a result of its external administration, in which case LH secretion is suppressed. The same negative feedback mechanisms operate when serum testosterone concentrations are reduced. Thus, if vitamin D were to significantly affect serum testosterone levels (at the gonadal or hypothalamic–pituitary level), it would have to characteristically modulate HPG activity, that is, testosterone and LH levels.

In our study, the LH levels were 5.09 ± 2.26 mIU/ml, 4.84 ± 2.29 mIU/ml, and 5.28 ± 2.83 mIU/ml in the groups with vitamin D levels of < 10 ng/ml, 10–20 ng/ml, and > 20 ng/ml, respectively. The results were not statistically different between the groups. The individual fractions of testosterone (total, free, and bioavailable) were also similar. As in the previous case, SHBG levels were not statistically significantly different in the homogeneous groups formed based on different vitamin D levels.

There are few studies in the available literature regarding the effects of vitamin D on steroidogenesis directly in the Leydig cells (usually animal experiments)^24,25^. Huang et al.^26^ showed that vitamin D does not increase testosterone synthesis in Leydig cells in the absence of LH, but it does increase synthesis of it when induced by LH in both immature and mature ram Leydig cells. This would confirm a potential role for vitamin D in Leydig cells. Holt et al.^27^ demonstrated the effect of 1,25(OH)_2_D_3_ on testosterone production in the male gonad. It appears that the stimulus effect may be at least partially direct. However, these results are not clear, as the authors pointed out that larger, placebo-controlled studies are needed to determine whether vitamin D supplementation can affect testosterone production.

Results similar to ours were obtained by Rudnicka et al.^28^, who showed no association between vitamin D levels and LH, FSH, TT, inhibin B, or estradiol levels (as well as semen parameters) in young men. In addition, Ramlau-Hansen et al.^29^ also found that LH, TT, and inhibin B levels were not significantly associated with vitamin D. Similarly, Lerchbaum et al.^30^ indicated that significantly elevated serum vitamin D levels in the subjects had no effect on changes in total testosterone levels in middle-aged men. Jorde et al.^31^ showed that supplementation with even high-dose vitamin D (20,000–40,000 IU/week for 6–12 months) does not lead to an increase in serum testosterone levels in healthy men.

Results contrary to ours were obtained by Tak et al.^32^, showing a statistically significant association between 25(OH)D level and total and free testosterone level in a group of 652 Korean men. However, it should be noted that these studies were performed in a rather heterogeneous group of men with multiple confounding factors. A similar situation occurred in the study by Rafiq et al.^33^, which investigated the relationship between vitamin D levels and various testesterone and SHBG fractions in a group of 459 men. The authors observed a statistically significant positive correlation between 25(OH)D level and total and bioavailable testosterone level. However, it should be emphasised that the study was conducted on a group of men aged 65–89 years, which reduces its cognitive value, for obvious reasons. Similarly, Lee et al.^34^ documented lower levels of FT and higher level of oestradiol and LH levels in 3369 men aged 40–79 years with vitamin D deficiency. The authors also observed that 25(OH)D was positively associated with TT, FT and negatively with oestradiol and LH in age- and centre-adjusted linear regressions. In addition, Chen et al.^35^ also showed an association between reduced vitamin D level and testosterone level in a group of 4524 men. The association between the study variables was not strong and the authors described it as a ‘biologically plausible causal effect.’ In contrast, Canguven et al.^36^ found that vitamin D supplementation (Ergocalciferol—Oral solution 600,000 IU/1.5 ml during 1 year) led to an increase in total testosterone level from 12.46 ± 3.30 to 15.99 ± 1.84 nmol/l. However, it is important to point out that this increase is not biologically meaningful. Furthermore, this study was conducted on middle-aged men (35–64 years).

Pliz et al.^37^ reported that vitamin D supplementation (3332 IU daily for 1 year) increased total, free, and bioavailable testosterone levels in a group of 31 middle-aged men (49.2 ± 10.2). The authors concluded that further studies are needed to confirm the hypothesis that vitamin D supplementation can increase testosterone level.

Currently, there are no studies that would explain the mechanism of the possible effects of vitamin D on increasing or decreasing testosterone synthesis. Suggestions that serum testosterone levels are due to the effects of vitamin D on Leydig cell calcium homeostasis, aromatase activity, osteocalcin, or the activity of extragenic vitamin D^6,9,38–41^ are preliminary and require further study.

When analysing the available literature, it is worth noting that many studies have been performed in groups of men in which various pathologies already existed at baseline. A frequently cited study by Blomberg-Jensen et al.^41^ was conducted in a group of men with fertility disorders, a group that may have already had baseline impaired endocrine or exocrine testicular function.

It should be emphasised that in our study the participants coming from Lower Silesia had normal BMI (24.0 ± 2.9), only about 14% declared that they smoked cigarettes, 90% declared that they consumed alcohol (beer, wine, or vodka in amounts corresponding to ≥ 50 g of ethanol per week), and the level of physical activity was high (≥ 3000 MET-min/week for 60.2% of the participants).

Differences reported in other studies that focussed on the relationship between vitamin D concentrations and sex hormone concentrations in young and older men may be due to the absence or presence of comorbidities related to age, lifestyle, and physical activity, which in turn may affect vitamin D and hormone levels.

## Limitation

Our study has some limitations. Firstly, the study group was small, so further research on a larger group of men is needed. Secondly, men from Lower Silesia showed significant insufficient and deficient 25(OH)D levels (n = 148, 83.5%); therefore, the study should be extended to include subjects with normal vitamin D levels. Thirdly, the subjects were a homogeneous group with a similar level of education, lifestyle, and especially physical activity.

## Conclusions

In conclusion, the results of our study demonstrate the lack of an association between deficient/insufficient 25(OH)D concentration and androgen levels in a group of young, healthy, physically active men living in an industrialised region of Poland. It should be noted that results of our study does not exclude possibility that implementation of the proper vitamin D supplementation protocol may affect levels of testosterone. Therefore, further research is necessary to verify effects of vitamin D supplementation on androgen levels in young, healthy men.

## References

[1] Palacios C, Gonzalez L (2014). Is vitamin D deficiency a major global public health problem?. J. Steroid. Biochem. Mol. Biol..

[2] Ceglia L, Harris SS (2013). Vitamin D and its role in skeletal muscle. Calcif. Tissue Int..

[3] Vondra K, Stárka L, Hampl R (2015). Vitamin D and thyroid diseases. Physiol. Res..

[4] Skaaby T, Thuesen BH, Linneberg A (2017). Vitamin D, cardiovascular disease and risk factors. Adv. Exp. Med. Biol..

[5] Prietl B, Treiber G, Pieber TR, Amrein K (2013). Vitamin D and immune function. Nutrients.

[6] de Angelis C, Galdiero M, Pivonellom C, Garifalos F, Menafra D, Cariati F (2017). The role of vitamin D in male fertility: A focus on the testis. Rev. Endocr. Metab. Disord..

[7] Bikle DD (2014). Vitamin D metabolism, mechanism of action, and clinical applications. Chem. Biol..

[8] Carlberg C (2014). Genome-wide (over)view on the actions of vitamin D. Front. Physiol..

[9] Blomberg Jensen M (2014). Vitamin D and male reproduction. Nat. Rev. Endocrinol..

[10] Lorenzen M, Boisen IM, Mortensen LJ, Lanske B, Juul A, Jensen MB (2017). Reproductive endocrinology of vitamin D. Mol. Cell Endocrinol..

[11] Skobowiat C, Dowdy JC, Sayre RM, Tuckey RC, Slominski A (2011). Cutaneous hypothalamic–pituitary–adrenal axis homolog: regulation by ultraviolet radiation. Am. J. Physiol. Endocrinol. Metab..

[12] Skobowiat C, Postlethwaite AE, Slominski AT (2017). Skin exposure to ultraviolet B rapidly activates systemic neuroendocrine and immunosuppressive responses. Photochem. Photobiol..

[13] Slominski A, Wortsman J (2000). Neuroendocrinology of the skin. Endocr. Rev..

[14] Slominski AT, Zmijewski MA, Skobowiat C, Zbytek B, Slominski RM, Steketee JD (2012). Sensing the environment: Regulation of local and global homeostasis by the skin's neuroendocrine system. Adv. Anat. Embryol. Cell Biol..

[15] Blomberg Jensen M, Nielsen JE, Jørgensen A, Rajpert-De Meyts E, Kristensen DM (2010). Vitamin D receptor and vitamin D metabolizing enzymes are expressed in the human male reproductive tract. Hum. Reprod..

[16] Mędraś M, Lwow F, Jóźków P, Szmigiero L, Zagrodna A, Zagocka E (2017). The quality of semen among a sample of young, healthy men from Lower Silesia (AndroLS). Endokrynol. Pol..

[17] Jóźków P, Mędraś M, Lwow F, Zagrodna A, Słowińska-Lisowska M (2017). Associations between physical activity and semen quality in young healthy men. Fertil. Steril..

[18] Jóźków P, Słowińska-Lisowska M, Zagrodna A, Mędraś M, Lwow F (2018). Vitamin D and semen quality IN urban, young, healthy men (ANDROLS). J. Mens. Health.

[19] Craig CL, Marshall AL, Sjöström M, Bauman AE, Booth ML, Ainsworth BE (2003). International physical activity questionnaire: 12-country reliability and validity. Med. Sci. Sports Exerc.

[20] Kent M (2007). The Oxford Dictionary of Sports Science and Medicine.

[21] Ainsworth BE, Haskell WL, Whitt MC, Irwin ML, Swartz AM, Strath SJ (2000). Compendium of physical activities: An update of activity codes and MET intensities. Med. Sci. Sports Exerc..

[22] Holick MF, Binkley NC, Bischoff-Ferrari HA, Gordon CM, Hanley DA, Heaney RP (2011). Endocrine Society. Evaluation, treatment, and prevention of vitamin D deficiency: An Endocrine Society clinical practice guideline. J. Clin. Endocrinol. Metab..

[23] Pludowski P, Holick MF, Grant WB, Konstantynowicz J, Mascarenhas MR, Haq A (2018). Vitamin D supplementation guidelines. J. Steroid. Biochem. Mol. Biol..

[24] Sonnenberg J, Luine VN, Krey LC, Christakos S (1986). 1,25-Dihydroxyvitamin D3 treatment results in increased choline acetyltransferase activity in specific brain nuclei. Endocrinology.

[25] Osmundsen BC, Huang HF, Anderson MB, Christakos S, Walters MR (1989). Multiple sites of action of the vitamin D endocrine system: FSH stimulation of testis 1,25-dihydroxyvitamin D3 receptors. J. Steroid. Biochem..

[26] Huang Y, Jin H, Chen J, Jiang X, Li P, Ren Y (2015). Effect of Vitamin D on basal and Luteinizing Hormone (LH) induced testosterone production and mitochondrial dehydrogenase activity in cultured Leydig cells from immature and mature rams. Anim. Reprod. Sci..

[27] Holt R, Juel Mortensen L, Harpelunde Poulsen K, Nielsen JE, Frederiksen H, Jørgensen N (2020). Vitamin D and sex steroid production in men with normal or impaired Leydig cell function. J. Steroid. Biochem. Mol. Biol..

[28] Rudnicka A, Adoamnei E, Noguera-Velasco JA, Vioque J, Cañizares-Hernández F, Mendiola J (2020). Vitamin D status is not associated with reproductive parameters in young Spanish men. Andrology.

[29] Ramlau-Hansen CH, Moeller UK, Bonde JP, Olsen J, Thulstrup AM (2011). Are serum levels of vitamin D associated with semen quality? Results from a cross-sectional study in young healthy men. Fertil. Steril..

[30] Lerchbaum E, Pilz S, Trummer C, Schwetz V, Pachernegg O, Heijboer AC (2017). Vitamin D and testosterone in healthy men: A randomized controlled trial. J. Clin. Endocrinol. Metab..

[31] Jorde R, Grimnes G, Hutchinson MS, Kjærgaard M, Kamycheva E, Svartberg J (2013). Supplementation with vitamin D does not increase serum testosterone levels in healthy males. Horm. Metab. Res..

[32] Tak YJ, Lee JG, Kim YJ, Park NC, Kim SS, Lee S (2015). Serum 25-hydroxyvitamin D levels and testosterone deficiency in middle-aged Korean men: A cross-sectional study. Asian J. Androl..

[33] Rafiq R, van Schoor NM, Sohl E, Zillikens MC, Oosterwerff MM, Schaap L (2016). Associations of vitamin D status and vitamin D-related polymorphisms with sex hormones in older men. J. Steroid. Biochem. Mol. Biol..

[34] Lee DM, Tajar A, Pye SR, Boonen S, Vanderschueren D, Bouillon R (2012). Association of hypogonadism with vitamin D status: The European Male Ageing Study. Eur. J. Endocrinol..

[35] Chen C, Zhai H, Cheng J, Weng P, Chen Y, Li Q (2019). Causal link between vitamin D and total testosterone in men: A Mendelian randomization analysis. J. Clin. Endocrinol. Metab..

[36] Canguven O, Talib RA, El Ansari W, Yassin DJ, Al Naimi A (2017). Vitamin D treatment improves levels of sexual hormones, metabolic parameters and erectile function in middle-aged vitamin D deficient men. Aging Male.

[37] Pilz S, Frisch S, Koertke H, Kuhn J, Dreier J, Obermayer-Pietsch B (2011). Effect of vitamin D supplementation on testosterone levels in men. Horm. Metab. Res.

[38] Kinuta K, Tanaka H, Moriwake T, Aya K, Kato S, Seino Y (2000). Vitamin D is an important factor in estrogen biosynthesis of both female and male gonads. Endocrinology.

[39] Haussler MR, Jurutka PW, Mizwicki M, Norman AW (2011). Vitamin D receptor (VDR)-mediated actions of 1α,25(OH)_2_ vitamin D_3_: Genomic and non-genomic mechanisms. Best Pract. Res. Clin. Endocrinol. Metab..

[40] Trummer C, Pilz S, Schwetz V, Obermayer-Pietsch B, Lerchbaum E (2018). Vitamin D, PCOS and androgens in men: A systematic review. Endocr. Connect.

[41] Blomberg Jensen M, Gerner Lawaetz J, Andersson AM, Petersen JH, Nordkap L, Bang AK (2016). Vitamin D deficiency and low ionized calcium are linked with semen quality and sex steroid levels in infertile men. Hum. Reprod..

